# Ultrasound-guided sacral multifidus plane block versus caudal epidural block for postoperative analgesia in pediatric hypospadias surgery: A randomized double-blind controlled trial

**DOI:** 10.1371/journal.pone.0353110

**Published:** 2026-07-30

**Authors:** Amr Sobhy, Radwa Tarek Mohamed, Gihan seif, Manal mohamed kamal shams, Adham Magdy Haggag, Abd El-Aziz A. Abd EL-Aziz

**Affiliations:** Department of Anesthesia, Intensive Care and Pain Management, Faculty of Medicine, Ain Shams University, Cairo, Egypt; Yarmouk University, JORDAN

## Abstract

**Introduction:**

Hypospadias repair is associated with moderate to severe postoperative pain in children. Caudal epidural block is traditionally considered the standard regional anesthetic technique for sub-umbilical pediatric surgeries; however, its duration of analgesia and potential adverse effects may be limited. The sacral multifidus plane block (SMPB) is a novel ultrasound-guided fascial plane technique that may provide prolonged analgesia with fewer complications. The aim of this study is to compare the analgesic efficacy and safety of ultrasound-guided sacral multifidus plane block versus caudal epidural block in children undergoing hypospadias repair.

**Materials and methods:**

This prospective, randomized, double-blind controlled trial included 30 children aged 1–5 years (ASA I–II) scheduled for hypospadias repair. Patients were randomly allocated to receive either SMPB (n = 15) or caudal block (n = 15), using 1 mL/kg of 0.25% bupivacaine. The primary outcome was time to first rescue analgesia. Secondary outcomes included postoperative FLACC pain scores, total rescue analgesic consumption, intraoperative fentanyl requirements, hemodynamic variables, and block-related complications. Pain was assessed for 24 hours postoperatively.

**Results:**

Demographic and intraoperative characteristics were comparable between groups. Time to first rescue analgesia was significantly longer in the SMPB group compared with the caudal group (14.8 ± 3.6 vs 9.3 ± 4.2 hours, p = 0.004). FLACC scores were significantly lower in the SMPB group at 6 and 12 hours postoperatively (p = 0.019 and p = 0.001, respectively). Total diclofenac consumption was lower in the SMPB group (0.40 ± 0.20 mg/kg vs 0.70 ± 0.24 mg/kg, p = 0.010). Intraoperative fentanyl requirements and hemodynamic parameters were similar. No major complications were observed.

**Conclusion:**

Ultrasound-guided sacral multifidus plane block provides longer-lasting postoperative analgesia and reduces rescue analgesic requirements compared with caudal epidural block in pediatric hypospadias surgery, with a comparable safety profile. SMPB may represent a reliable alternative to caudal block for postoperative pain management in this population.

## Introduction

Hypospadias is one of the most common congenital anomalies of the male external genitalia, characterized by abnormal ventral placement of the urethral meatus resulting from incomplete development and fusion of the urethral folds. It is second in prevalence only to cryptorchidism and typically requires surgical correction in early childhood to achieve optimal functional and cosmetic outcomes. Despite advances in surgical techniques, postoperative pain following hypospadias repair remains a significant clinical concern [1].

Children undergoing hypospadias surgery experience moderate to severe perioperative pain due to extensive manipulation of highly innervated penile and perineal tissues. Inadequate analgesia in this population is associated with adverse physiological stress responses, delayed recovery, increased postoperative complications, heightened parental anxiety, and the potential development of maladaptive pain behaviors. Consequently, effective perioperative pain control is a cornerstone of pediatric urological anesthesia [2].

Systemic opioids have traditionally been used for postoperative analgesia; however, their use in children is limited by well-known adverse effects including respiratory depression, nausea, vomiting, pruritus, urinary retention, and sedation. These concerns have driven growing interest in regional anesthesia techniques as part of multimodal analgesic strategies aimed at improving analgesic quality while minimizing opioid exposure [3].

Caudal epidural block has long been regarded as the standard regional anesthetic technique for sub-umbilical surgeries in children. It is relatively simple to perform, has a high success rate, and provides reliable intraoperative and early postoperative analgesia. Nevertheless, caudal block may be associated with motor blockade, urinary retention, limited duration of action, and variable spread of local anesthetic, particularly when performed without ultrasound guidance. These limitations have prompted exploration of alternative regional techniques that may offer longer lasting and more targeted analgesia [4–6].

Recent advances in ultrasound technology have facilitated the development of fascial plane blocks, which allow visualization of relevant anatomy, accurate needle placement, and real-time monitoring of local anesthetic spread. Among these, the erector spinae plane block has gained widespread attention, and its sacral modifications have been investigated for surgeries involving sacral and perineal dermatomes [7].

The sacral multifidus plane block (SMPB) is a relatively novel ultrasound-guided interfascial plane block in which local anesthetic is deposited in the plane deep to the sacral multifidus muscle. This technique is anatomically distinct from the sacral erector spinae plane block and is thought to provide multisegmental sensory blockade of the dorsal rami of sacral spinal nerves, potentially resulting in prolonged analgesia with minimal motor or autonomic involvement [8].

Although preliminary studies suggest that sacral plane blocks may offer effective analgesia for pediatric perineal surgery, high-quality randomized trials directly comparing SMPB with caudal epidural block are scarce. Therefore, this randomized double-blind controlled trial was designed to compare the analgesic efficacy and safety of ultrasound-guided sacral multifidus plane block versus caudal epidural block in children undergoing hypospadias repair.

The primary outcome was time to first rescue analgesia. Secondary outcomes included postoperative FLACC pain scores, total rescue analgesic consumption, intraoperative fentanyl requirements, hemodynamic parameters, and block-related complications

## Materials and methods

### Study design and ethical approval

This prospective randomized double-blind controlled trial was conducted at Ain Shams University Hospitals, Cairo, Egypt, between December 2024 and April 2025. The study protocol was approved by the Research Ethics Committee of the Faculty of Medicine, Ain Shams University (approval number: FAMASU R268/2024). Written informed consent was obtained from the parents or legal guardians of all participants. The trial was registered at ClinicalTrials.gov (NCT06561269) and conducted according to the Declaration of Helsinki and CONSORT 2010 guidelines.

### Participants

Children aged 1–5 years with American Society of Anesthesiologists (ASA) physical status I or II scheduled for elective hypospadias repair were eligible for inclusion.

Exclusion criteria were refusal of parental consent, congenital spinal anomalies, developmental delay or altered mental status, coagulation disorders or anemia, infection at the injection site, and known allergy to study medications.

### Sample size calculation

Based on previous study (Ozen and Dogakan 2020) [9], the pooled standard deviation of time to first rescue analgesia is calculated to be 3.26 hours. Sample size calculation revealed that at least 15 patients in each group are needed to detect a minimal detectable difference of 4 hours, using unpaired t-test, assuming the calculated pooled SD, significant level of 0.05, and a power of 0.9 using power analysis and sample size software (PASS15) (Version 15.0.10)

### Randomization and blinding

Patients were randomized using computer-generated random number sequences into two equal groups: sacral multifidus plane block group (SMPB) and caudal block group (CB). Allocation concealment was achieved using sealed opaque envelopes. Patients, parents, surgeons, and investigators responsible for data collection and postoperative assessment were blinded to group assignment. The anesthesiologist performing the block was not involved in outcome assessment.

### Anesthetic management

Standard fasting guidelines were followed. On arrival in the operating room, baseline heart rate, noninvasive blood pressure, electrocardiography, and peripheral oxygen saturation were recorded.

General anesthesia was induced with sevoflurane (4–8%) in 80% oxygen via face mask, followed by intravenous cannulation. Children aged 1–4 years received fentanyl 1 µg/kg, and neuromuscular blockade was achieved with atracurium 0.5 mg/kg. Children aged 4–5 years received fentanyl 1 µg/kg and propofol 1–2 mg/kg prior to atracurium administration. Anesthesia was maintained with isoflurane 1–2% in oxygen.

Following stabilization of hemodynamic parameters and before surgical incision, the assigned regional block was performed.

### Block techniques

All blocks were performed under strict aseptic precautions using a high-frequency linear ultrasound probe (2.5–7.5 MHz).

### Sacral multifidus plane block group

With the patient in the prone position, the ultrasound probe was placed in the midline over the sacrum to identify the multifidus muscle and the median sacral crests at the S2–S3 level. Using an in-plane technique, a block needle was advanced to the fascial plane deep to the multifidus muscle. A volume of 1 mL/kg of 0.25% bupivacaine was injected after negative aspiration.

### Caudal block group

With the patient in the lateral decubitus position, the sacral hiatus was identified using ultrasound in transverse and longitudinal views. Using an in-plane approach, the needle was inserted into the caudal epidural space and 1 mL/kg of 0.25% bupivacaine was injected after negative aspiration.

After block performance, patients were positioned supine and received intravenous Ringer’s solution at 10 mL/kg/h. Surgical incision was allowed no sooner than 20 minutes after block administration (to allow adequate onset and stabilization of the regional block before surgical incision and to ensure standardized assessment of block efficacy under consistent anesthetic conditions).

### Intraoperative monitoring

Heart rate, mean arterial pressure, and oxygen saturation were recorded immediately after block placement, every 5 minutes for the first 15 minutes, and then every 15 minutes until the end of surgery.

If heart rate or blood pressure increased by more than 20% from baseline, fentanyl 1 µg/kg was administered as rescue analgesia.

### Postoperative analgesia and assessment

Pain was evaluated using the Face, Legs, Activity, Cry, Consolability (FLACC) scale by a blinded observer at recovery and at 1, 2, 4, 6, 12, 18, and 24 hours postoperatively.

For FLACC score ≥3, diclofenac suppository 0.5 mg/kg was administered as first-line rescue analgesic. If pain persisted or recurred, intravenous paracetamol 15 mg/kg was administered as second-line rescue.

Hemodynamic parameters and respiratory rate were recorded in the post-anesthesia care unit at 0, 15, 30, and 60 minutes, then hourly for 4 hours, and every 4 hours thereafter for 24 hours.

Adverse events including nausea, vomiting, urinary retention, hypotension, hematoma, nerve injury, infection, and respiratory depression were documented.

### Outcome measures

The primary outcome was time to first rescue analgesia.

Secondary outcomes included postoperative FLACC scores, total rescue analgesic consumption, intraoperative fentanyl requirements, hemodynamic parameters, and incidence of complications.

### Statistical analysis

Data were analyzed using SPSS software version 23. Quantitative variables were first assessed for normality using the Shapiro–Wilk test, together with visual inspection of histograms and Q–Q plots. Normally distributed continuous variables were summarized as mean ± standard deviation and compared between groups using the independent-samples t-test. Non-normally distributed or ordinal variables were summarized as median and interquartile range and compared using the Mann–Whitney U test. Categorical variables were presented as number and percentage and compared using the chi-square test or Fisher’s exact test when expected cell counts were small.The primary outcome was time to first rescue analgesia Using Independent-samples t-test for normally distributed continuous variables. Mean difference is presented as MPB − Caudal with 95% CI. p-value >0.05 is insignificant; *p < 0.05 significant; **p < 0.01 highly significant. All participants experienced the event (first rescue analgesia) within the 24-hour observation period; therefore, no censoring occurred and survival analysis methods were not required. Secondary outcomes included postoperative FLACC scores performed using a repeated-measures mixed-effects model, Repeated postoperative pain scores and hemodynamic measurements were reanalyzed using repeated-measures ANOVA/mixed-effects methodology to appropriately account for within-subject correlations over time. To minimize inflation of type I error, Bonferroni correction was applied for repeated post hoc comparisons across time points.

## Results

### Participant flow and baseline characteristics

**Fig 1:**
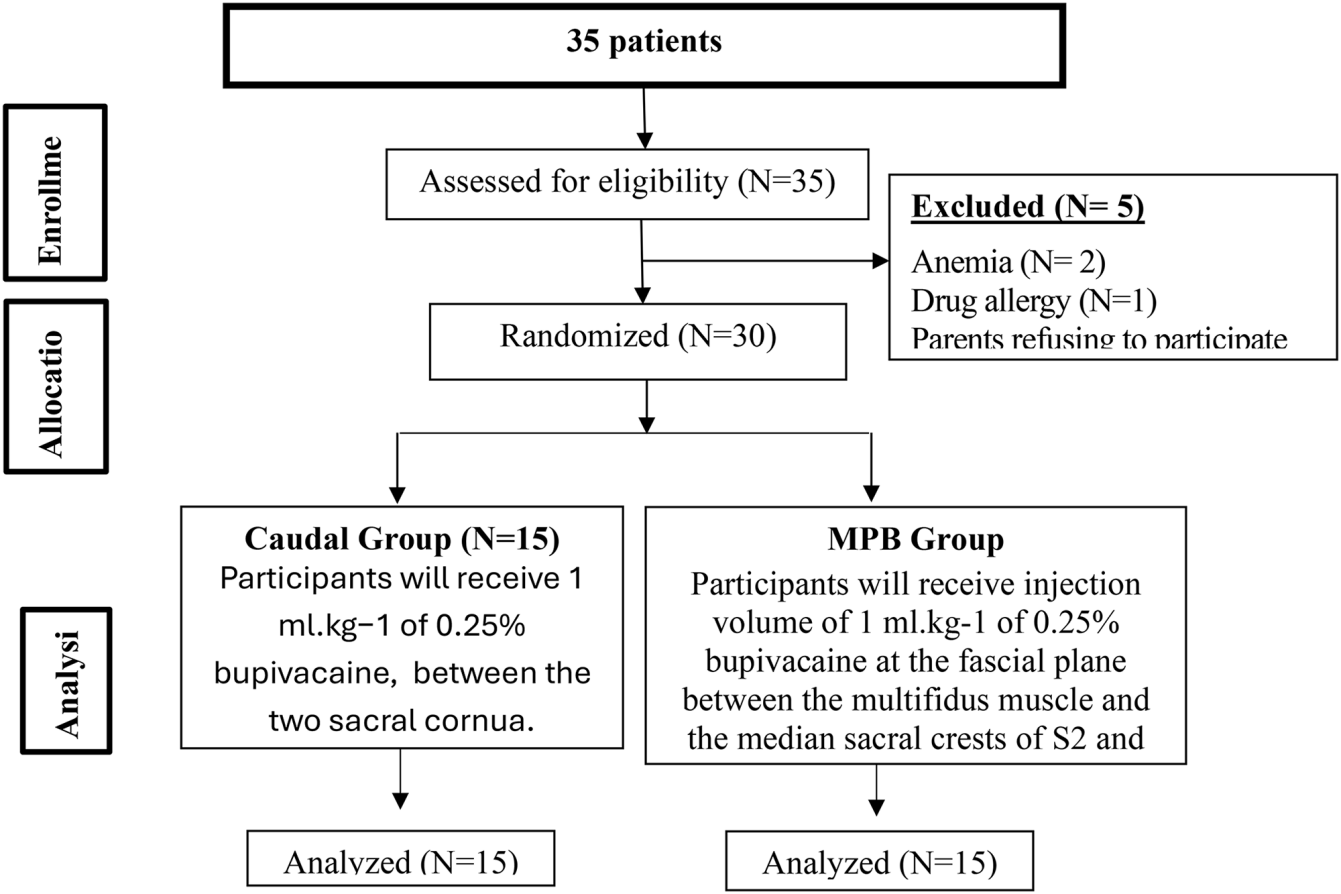
Study consort chart. Thirty-five children were assessed for eligibility; five were excluded (two with anemia, one with drug allergy, and two due to parental refusal). Thirty patients were randomized and equally allocated to the sacral multifidus plane block (SMPB) group (n = 15) or caudal block (CB) group (n = 15). All randomized patients completed the study and were included in the final analysis.

Participant flow enrolled in Fig 1. Baseline demographic and clinical characteristics were comparable between groups. There were no significant differences in age, weight, or ASA physical status, indicating successful randomization and homogeneity of the study population (p > 0.05) in Table 1.

**Table 1:** Demographic characteristics of the studied groups.

		Caudal group (n = 15)		MPB group (n = 15)		Effect estimate	95% CI	P-value
		N	%	N	%			
**Age** **(Year)**	**Mean ±SD**	3.60 ± 1.12		4.10 ± 1.18		MD = 0.47	−0.17 to 1.11	0.147
	**Range**	3–5		3–5				
**Weight** **(Kg)**	**Mean ±SD**	18.60 ± 2.88		18.85 ± 4.22		MD = 0.21	−2.50 to 2.92	0.872
	**Range**	15–23		14–25				
**ASA**	**I**	12	80.0	13	86.7	RD = 6.7%	−21.1% to 33.6%	1.000
	**II**	3	20.0	2	13.3	RD = −6.7%	−33.6% to 21.1%	1.000

Using: Independent-samples t-test for normally distributed continuous variables and Fisher’s exact test for categorical variables. Effect estimates are presented as MPB − Caudal with 95% CI. p-value >0.05 is insignificant; *p < 0.05 significant; **p < 0.01 highly significant.

### Intraoperative characteristics

Mean intraoperative fentanyl consumption was similar between the SMPB and CB groups with a mean difference of 0.21 mcg, 95% CI −2.48 to 2.91, p = 0.872. Duration of anesthesia (56.1 ± 5.35 vs 54.6 ± 4.90 minutes; p = 0.220) However, surgery duration was slightly longer in the MPB group, with a mean difference of 2.53 minutes, 95% CI 0.34 to 4.72, p = 0.025, although this small difference is unlikely to be clinically meaningful. No patient required additional intraoperative fentanyl beyond the standardized induction dose Table 2.

**Table 2:** Timing of procedure among the studied groups.

		Caudal group (n = 15)	MPB group (n = 15)	Mean difference	95% CI	P-value
**Total Fentanyl (mic)**	**Mean ±SD**	18.60 ± 2.88	18.85 ± 4.22	0.21	−2.48 to 2.91	0.872
	**Range**	15–23	14–25			
**Duration of Anesthesia (min)**	**Mean ±SD**	54.6 ± 4.90	56.1 ± 5.35	1.53	−0.97 to 4.04	0.220
	**Range**	50–60	50–60			
**Duration of Surgery (min)**	**Mean ±SD**	**49.6 ± 4.71**	**52.2 ± 4.07**	**2.53**	**0.34 to 4.72**	0.025*
	**Range**	**45–55**	**45–55**			

Using: Independent-samples t-test for normally distributed continuous variables. Mean difference is presented as MPB − Caudal with 95% CI. p-value >0.05 is insignificant; *p < 0.05 significant; **p < 0.01 highly significant.

### Hemodynamic and respiratory parameters

Heart rate, systolic blood pressure, diastolic blood pressure, and oxygen saturation remained stable throughout the intraoperative period in both groups. No statistically significant differences were observed at any recorded time point (p > 0.05). No episodes of clinically significant hypotension, bradycardia, or desaturation were recorded.

### Postoperative pain scores

**Fig 2:**
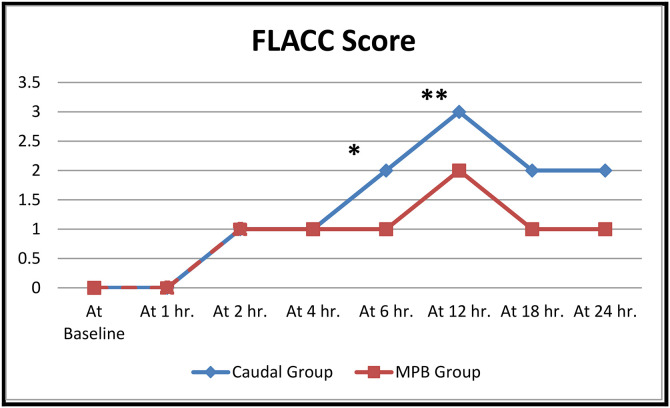
FLACC pain score among the Studied Groups over Time. Postoperative Face, Legs, Activity, Cry, Consolability FLACC pain scores were low and comparable between groups in the early postoperative period (baseline to 2 hours). At 6 hours, the SMPB group demonstrated significantly lower FLACC scores compared with the CB group (median 1 [IQR 0–2] vs 1[IQR 1–3]; p = 0.001,95%CI −1.46 to −0.41). The greatest difference was observed at 12 hours, where MPB reduced the FLACC score by 1.27 points compared with caudal block, 95% CI −1.79 to −0.74, p < 0.001. These findings suggest a more sustained analgesic effect with MPB during the first 24 postoperative hours.

Postoperative FLACC pain scores were low and comparable between groups in the early postoperative period (baseline to 2 hours). At 6 hours, the SMPB group demonstrated significantly lower FLACC scores compared with the CB group (median 1 [IQR 0–2] vs 1[IQR 1–3]; p = 0.001,95%CI −1.46 to −0.41). The greatest difference was observed at 12 hours, where MPB reduced the FLACC score by 1.27 points compared with caudal block, 95% CI −1.79 to −0.74, p < 0.001. These findings suggest a more sustained analgesic effect with MPB during the first 24 postoperative hours (Table 3, Fig 2).

**Table 3:** FLACC pain score among the studied groups over time.

FLACC		Caudal Group (n = 15)	MPB Group (n = 15)	Mean difference	95% CI	P-value
**At Baseline**	**Median (IQR)**	0 (0–1)	0 (0–1)	0	−0.53 to 0.53	1.000
	**Range**	0–2	0–2			
**At 2 hr.**	**Median (IQR)**	1 (0–1)	1 (0–1)	0.07	−0.46 to 0.59	0.804
	**Range**	0–2	0–2			
**At 4 hr.**	**Median (IQR)**	**1 (1– 2)**	**1 (0–1)**	−0.53	−1.06 to −0.01	0.047
	**Range**	**1–2**	**0–2**			
**At 6 hr.**	**Median (IQR)**	**2 (1–3)**	**1 (0–2)**	**−0.93**	**−1.46 to −0.41**	0.001**
	**Range**	**1–4**	**0–2**			
**At 12 hr.**	**Median (IQR)**	**3 (2 –4)**	**2 (1–2)**	**−1.27**	**−1.79 to −0.74**	<0.001**
	**Range**	**2–5**	**0–3**			
**At 18 hr.**	**Median (IQR)**	**2 (1 –3)**	**1 (0–2)**	**−0.93**	**−1.46 to −0.41**	0.001**
	**Range**	**1–4**	**0–3**			
**At 24 hr.**	**Median (IQR)**	**2 (1–2)**	**1 (0–2)**	**−0.73**	**−1.26 to −0.21**	0.007**
	**Range**	**1–2**	**0–2**			
**Mean Change**		**1.40 ± 0.63**	**0.67 ± 0.62**	**−0.73**	**−1.20 to −0.27**	0.003**
**Within Group P-value**		<0.001**	0.001**			

Using: FLACC score was summarized as median (IQR) because it is ordinal/non-normal. Longitudinal analysis was performed using a repeated-measures mixed-effects model. Mean difference is presented as MPB − Caudal with 95% CI.. p-value >0.05 is insignificant; *p < 0.05 significant; **p < 0.01 highly significant.

Group effect: p=0.008 Time effect: p<0.001*

Group × time interaction: p < 0.001**

the Bonferroni-corrected significance threshold for the seven repeated FLACC time points is α = 0.05/7 = 0.007. All pairwise between-group comparisons at individual time points that were reported as significant (4 hr: p = 0.047; 6 hr: p = 0.001; 12 hr: p < 0.001; 18 hr: p = 0.001; 24 hr: p = 0.007)

### Rescue analgesic requirements

Time to first rescue analgesia was significantly longer in the SMPB group compared with the CB group (14.8 ± 3.6 hours vs 9.3 ± 4.2 hours; p = **0.007, 95%CI: 2.01 to 11.33**).

Total diclofenac consumption during the first 24 postoperative hours was significantly lower in the SMPB group (0.40 ± 0.20 mg/kg) than in the CB group (0.70 ± 0.24 mg/kg; p = **0.004, 95%CI: −0.69 to −0.15**). No patient in either group required second-line rescue analgesia with intravenous paracetamol. These findings indicate that MPB provided more durable postoperative analgesia and reduced rescue analgesic requirements during the first 24 postoperative hours (Table 4).

**Table 4:** Rescue patterns and diclofenac consumption among the studied groups.

		Caudal Group (n = 15)	MPB Group (n = 15)	Mean Difference	95% CI	P-value
**Time to First Analgesic Request (hr.)**	**Mean ±SD**	**9.30 ± 4.21**	**14.8 ± 3.62**	**6.67**	**2.01 to 11.33**	0.007**
	**Range**	**6–12**	**10–18**			
**Total Diclofenac Dose (mg/Kg)**	**Mean ±SD**	**0.7 ± 0.24**	**0.40 ± 0.20**	**−0.42**	**−0.69 to −0.15**	0.004**
	**Range**	**0–2**	**0–1**			

Using: Time-to-event analysis for time to first rescue analgesia, with patients not requiring rescue analgesia censored at 24 hours. Mean difference is presented as MPB − Caudal with 95% CI. p-value >0.05 is insignificant; *p < 0.05 significant; **p < 0.01 highly significant.

### Postoperative complications

See Fig 3.

**Fig 3:**
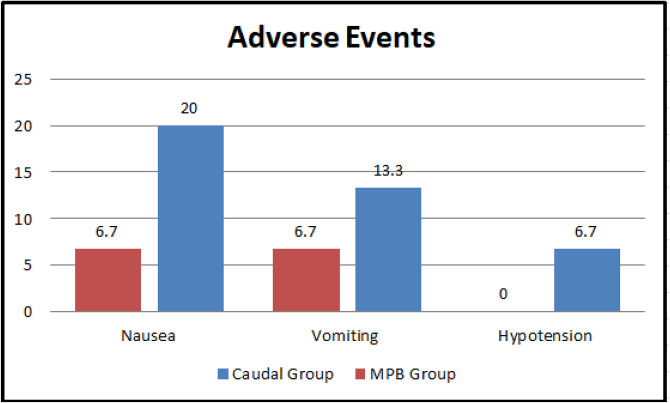
Adverse events among the studied groups. The overall incidence of complications was low in both groups. Nausea and vomiting occurred more frequently in the CB group (20% and 13.3%, respectively) compared with the SMPB group (6.7% and 6.7%). Urinary retention and hypotension were observed only in the CB group (13.3% and 6.7%, respectively). No cases of hematoma, nerve injury, infection, or respiratory depression were reported in either group. Differences between groups were not statistically significant.

## Discussion

This randomized double-blind controlled trial compared the analgesic efficacy and safety of ultrasound-guided sacral multifidus plane block (SMPB) with caudal epidural block in children undergoing hypospadias repair. The principal finding of this study is that SMPB provided significantly longer-lasting postoperative analgesia, lower pain scores during the mid-postoperative period, and reduced rescue analgesic consumption compared with caudal block, while maintaining comparable intraoperative analgesia and hemodynamic stability. These results support SMPB as an effective and reliable alternative to caudal block for pediatric hypospadias surgery.

Baseline demographic characteristics and operative durations were similar between groups, confirming appropriate randomization and minimizing potential confounding factors. Intraoperative fentanyl requirements were similar between groups (18.6 µg in caudal vs 18.9 µg in SMPB, p = 0.942), and no patient required supplemental opioid administration, indicating that both techniques provided adequate intraoperative analgesia when combined with general anesthesia. These findings are consistent with previous studies demonstrating that both caudal and sacral plane blocks effectively attenuate nociceptive responses during pediatric urological surgery [10^–^11].

Hemodynamic parameters and oxygen saturation remained stable throughout the intraoperative period in both groups, with no clinically significant differences. The absence of major cardiovascular disturbances reinforces the safety of both techniques. Notably, hypotension was observed only in the caudal group, although the difference was not statistically significant. This trend may be explained by greater sympathetic blockade associated with epidural spread following caudal injection, whereas SMPB primarily produces a sensory block with minimal autonomic involvement [11,12].

The primary outcome of this study, the mean time to first analgesic request was significantly prolonged in the SMPB group (14.8 ± 3.6 hours vs 9.3 ± 4.2 hours; p = 0.007, 95%CI: 2.01 to 11.33), confirming that SMPB provides more durable postoperative analgesia. This finding is consistent with recent systematic reviews and meta-analyses demonstrating that sacral multifidus and erector spinae plane blocks provide effective and prolonged analgesia for perineal and pelvic procedures [8].

Patients in the SMPB group required significantly less total rescue diclofenac (0.70 ± 0.24 mg/kg; p = 0.004, 95%CI: −0.69 to −0.15), and none required second-line rescue with paracetamol. This reduction in analgesic consumption is clinically significant, as it may translate into fewer opioid-related side effects, reduced nursing workload, and improved patient and parental satisfaction [13].

The ability to achieve prolonged analgesia with a single-shot block, without the need for catheter placement or continuous infusion, represents a practical advantage for SMPB in ambulatory and day-case surgical settings [14,15].

The secondary outcome of this study postoperative pain as assessed by FLACC scores—revealed a significant advantage for SMPB. Although both groups demonstrated low pain scores in the immediate postoperative period, SMPB patients exhibited significantly lower pain scores at At 6 hours, the SMPB group demonstrated significantly lower FLACC scores compared with the CB group (p = 0.001,95%CI −1.46 to −0.41) The greatest difference was observed at 12 hours (p = 0.001, 95%CI-1.79 to −0.74) compared to caudal block. By 18 and 24 hours, the difference was no longer statistically significant, suggesting that the analgesic advantage of SMPB is most pronounced during the critical early-to-mid postoperative period when pain intensity is typically highest. These findings are in line with the randomized controlled trial by **Bansal et al.,** [16] found that sacral ESPB more than doubled the time to first rescue analgesia compared with caudal block (21.3 vs 9.4 h), with lower FLACC scores and reduced paracetamol use in the ESPB group.

Although our findings suggest the superiority of the sacral multifidus plane block, this is not a universal consensus. Most notably, a 2025 randomized controlled trial by **Demirci et al**. [17] directly contradicted our results, reporting that caudal block provided a significantly longer duration of analgesia (9.7 hours vs. 6.3 hours) and lower postoperative FLACC scores compared to sacral erector spinae plane block in children undergoing hypospadias repair

In a notable contrast, a 2025 randomized trial by **Khan, Rafiq, and Yousaf** [18] directly comparing sacral ESP block with caudal block in children undergoing hypospadias repair found that caudal block provided a significantly longer duration of analgesia (mean 14.6 hours) compared to their sacral ESP block group (mean 10.2 hours). Furthermore, their study reported lower FLACC pain scores in the caudal group during the first 8 hours postoperatively, suggesting superior early pain control with the traditional technique. This discrepancy may be attributable to methodological variations between studies, such as differences in local anaesthetic volume, concentration, or the use of adjuvants like dexmedetomidine, which can significantly influence the duration and quality of both block types. Therefore, while our data and several other studies support the prolonged efficacy of sacral plane blocks, the evidence is not entirely uniform, indicating that procedural and pharmacological details are critical determinants of the final analgesic outcome.

Similarly, a recent meta-analysis by **Masiero et al.** [19] reviewing pediatric abdominal and sub-abdominal surgeries blocks concluded that sacral plane blocks and caudal blocks are often similarly effective, finding no statistically significant difference in early pain scores between the two techniques. These discrepancies suggest that the ‘superiority’ of sacral plane blocks may depend heavily on specific technical factors—such as the precise volume of local anesthetic injected or the craniocaudal spread achieved—rather than being an inherent advantage of the technique itself. The anatomical basis for this prolonged effect may relate to the multi-segmental spread of local anesthetic within the fascial plane beneath the multifidus muscle, targeting the dorsal rami of sacral spinal nerves and potentially achieving broader sensory coverage than single-point caudal injection [20].

The overall complication rate was low in both groups, reflecting the inherent safety of ultrasound-guided regional techniques in pediatric anesthesia which is consistent with [21]. However, a notable trend emerged: urinary retention occurred in two caudal patients (13.3%) and none in the SMPB group. Although this difference did not reach statistical significance, it is consistent with previous literature reporting that caudal block can interfere with bladder function due to sacral parasympathetic blockade, particularly when higher concentrations of local anesthetic or adjuncts are used [10–22]. For instance, the large RCT by Singh et al. [23] reported no significant difference in the incidence of nausea, vomiting, or other minor complications between their sacral ESPB and caudal groups, with both techniques being exceptionally well-tolerated and free of major adverse events in pediatric patients undergoing hypospadias repair The safety profile of SMPB in this study was favorable [24].

From a clinical perspective, SMPB offers several practical advantages. It provides prolonged analgesia with a single injection, avoids entry into the epidural space, and may facilitate faster recovery and improved comfort in the postoperative period. These characteristics align well with enhanced recovery after surgery (ERAS) principles, which emphasize opioid-sparing multimodal analgesia and early mobilization [1,25].

## Limitations

This study has limitations. The sample size was relatively small and may not have been sufficient to detect differences in rare complications. The single-center design may limit generalizability. Follow-up was restricted to 24 hours, and longer-term outcomes such as patient satisfaction, time to ambulation, and chronic pain were not evaluated. Additionally, sensory and motor block characteristics were not objectively assessed. Finally, all blocks were performed by experienced anesthesiologists; the learning curve and success rate of SMPB in less experienced hands require further investigation

### Future directions

Larger multicenter randomized trials are warranted to confirm these findings and further define the role of SMPB in pediatric urological surgery. Future studies should explore optimal dosing, the use of adjuvants, and comparisons with other regional techniques such as pudendal or penile nerve blocks

## Conclusion

Ultrasound-guided sacral multifidus plane block provides superior and longer-lasting postoperative analgesia compared with caudal epidural block in children undergoing hypospadias repair. SMPB significantly prolongs the time to first rescue analgesia, reduces postoperative pain scores during the mid-postoperative period, and decreases rescue analgesic consumption, while maintaining stable hemodynamics and a comparable safety profile. These findings support SMPB as an effective, opioid-sparing, and potentially safer alternative to caudal block for postoperative pain management in pediatric hypospadias surgery.

## Supporting information

S1 File Protocol. (PDF)

S2 File CONSORT checklist. (PDF)
