# Supplementary material for: Ultrasound-guided sacral multifidus plane block versus caudal epidural block for postoperative analgesia in pediatric hypospadias surgery: A randomized double-blind controlled trial
Source: PLoS One. 2026 Jul 30;21(7):e0353110. doi: 10.1371/journal.pone.0353110 (PMC13422836; doi:10.1371/journal.pone.0353110)
Supplement: S1 File — (PDF) [file pone.0353110.s001.pdf]

Title of the Protocol: Ultrasound-guided Multifidus plane block Versus Ultrasound-guided Caudal block for Pediatric hypospadias surgery: a randomized controlled clinical trial.

Postgraduate Student: Radwa Tarek Mohamed Mahmoud

Degree: Master, Faculty of Medicine, Ain Shams University

DIRECTOR: Prof. Gihan Seif EL Nasr Mohamed
Academic Position: Professor of Anesthesiology
Intensive Care and Pain Management
Department: Anesthesiology, Intensive Care and Pain Management

Co-DIRECTOR: Prof. Manal Mohamed Kamal
Academic Position: Professor of Anesthesiology,
Intensive Care and Pain Management
Department: Anesthesiology, Intensive Care and Pain Management

Co-DIRECTOR: Dr. Abdelaziz Abdallah Abdelaziz
Academic Position: Assistant Professor of Anesthesiology, Intensive Care and Pain Management
Department: Anesthesiology, Intensive Care and Pain Management

Co-DIRECTOR: Dr. Amr Sobhy Abdelkway Adar
Academic Position: Assistant Professor of Anesthesiology, Intensive Care and Pain Management
Department: Anesthesiology, Intensive Care and Pain Management

Co-DIRECTOR: Dr. Adham Magdy Haggag
Academic Position: Lecturer of Anesthesiology, Intensive Care and Pain Management
Department: Anesthesiology, Intensive Care and Pain Management

What is already known on this subject? AND

What does this study add?

Peri-operative pain management in pediatric surgeries is considered a challenging issue for the anesthesiologists. The usage of regional anesthesia in association with general anesthesia (GA) is a simple intraoperative way that reduces (GA) requirements and side effects. Recently, paraspinal fascial plane block, the sacral multifidus plane block (MPB), was used successfully in infants. The study has been designed to determine the effect of MPB versus caudal epidural block to control peri-operative pain in hypospadias surgeries in children.

1. INTRODUCTION/ REVIEW

Hypospadias is the second most common birth defect in boys after cryptorchidism. It is characterized by incomplete development of the urethral fold and the ventral foreskin of the penis that causes abnormal positioning of the urethral opening that needs surgical intervention under general anesthesia. **(Donaire and Mendez, 2023)**

The hypospadias surgery is known to be associated with acute sever pain and requires potent analgesia post-surgical for long term, Despite the prodigious advance in the knowledge of mechanisms of acute post-operative pain, The effective and safe analgesic methods is still under research especially for children**. (Elbadry *et al.*, 2023)**

Since opioids are associated with respiratory depression, severe sedation, disorientation, constipation and urinary retention, there is high incidence of opiophobia with consequent inappropriate dosage prescription, particularly in the pediatric population.**(Mahajan *et al.*, 2021)**

Regional anesthesia is the infiltration of a peripheral nerve with an anesthetic agent and blocking transmission to avoid or relieve perioperative pain. Regional anesthesia can be used in coincidence with general anesthesia, postprocedural, and often for many acute and chronic pain conditions. **(Folino and Mahboobi, 2023)**

A variety of peripheral and central nerve blocks have been developed as a regional anesthesia in children to ensure that perioperative pain can be effectively controlled in a safe way, chief among them is single-injection caudal block, The first author to describe caudal anesthesia for children was Meredith Campbell in 1933. **(Campbell, 1933; Wiegele, Marhofer and Lönnqvist, 2019)**

Caudal blocks have been proved to be effective for perioperative analgesia for painful sub-umbilical surgeries, it is safe, simple, and has a high success rate. However, the main disadvantage is its limited duration of action. **(Hassan, Hassan and Elmetwally, 2018)**

The erector spinae plane block (ESPB) is a new paraspinal plane block first described in 2016 to relieve thoracic pain, then described for the first time for sacral dermatomes for a pilonidal sinus surgery by Tulgar et al. in 2019. **(Forero *et al.*, 2016; Tulgar *et al.*, 2019)**

Recently, a paraspinal fascial plane block, the sacral multifidus plane block (SMPB), has been reportedly used successfully in infants. SMPB is technically similar but anatomically different from ESPB where two separate injections of local anesthetic occur which targets the fascial plane underneath sacral multifidus muscle just laterally to the midline.**(*Sacral Multifidus Plane/Sacral ESP Block — BABY BLOCKS*, no date)**

The possible mechanism of action of SMPB includes blocking the dorsal rami and medial cluneal nerves directly by local anesthetic (LA) deposition and ventral rami by anterior LA spread through dorsal and ventral sacral foramina. The SMPB may also block the pudendal nerve (S2–S4), lumbosacral plexus, and sciatic nerve via the anterior and cranial LA spread.**(Mistry *et al.*, 2022)**

Aksu and Gurkan first describe the longitudinal midline approach and it's ability to provide effective analgesia for an infant undergoing hypospadias repair, and additionally hypothesized that epidural spread may occur, though the mechanism of this is not well elucidated.**(Aksu and Gürkan, 2020)**

The sacral multifidus plane block is considered a promising block in neonates for peri-operative analgesia. However, no data exists comparing caudal and sacral multifidus plane blocks in pediatric populations. **(Mahajan *et al.*, 2021)**

2. AIM/ OBJECTIVES

This study aims to evaluate the safety and efficacy of ultrasound-guided sacral multifidus plane block and compare its analgesic effect to ultrasound-guided caudal block in pediatric patients undergoing hypospadias surgery under general anesthesia. The primary outcome of the study is to compare the time for the first time to rescue analgesia postoperatively in the two groups. The secondary outcome is to compare the two blocks as regard; hemodynamic parameters, the total intraoperative IV fentanyl requirements, postoperative FLACC pain score and complications.

3. METHODOLOGY:

Patients and Methods

- Type of Study: Randomized controlled clinical trial.
- Study Setting: Ain-shams University Hospitals, Cairo, Egypt.
- Study Period: 6 months -1 year from approval of ethical committee.
- Study Population: Pediatric patients undergoing Hypospadias surgery.
- Sample size: By using Power Analysis and Sample Size (PASS 15)(Version 15.0.10) for sample size calculation, setting power at 99%, alpha error 0.05 and after reviewing previous study results (Ozen and Yigit, 2020) showing that among Pediatric Patients underwent hypospadias surgery, the median time of first rescue analgesics was lower in those took caudal epidural block than those took ultrasound-guided dorsal penile nerve block (14[12-17] versus 20.50 [20-21]respectively); Based on that and after considering 20% dropout rate, a sample size of at least 30 pediatric patients undergoing hypospadias surgery divided randomly into 2 groups (15 patients in each group) and will be sufficient to achieve study objective.

Inclusion Criteria:

- Children aged (1 –7) years old.
- All patients will be of ASA classification I, II physical status.

Exclusion Criteria:

- Parents refusing to participate are excluded from the trial.
- Children who had spinal anomalies, altered mental status.
- A history of developmental delay.
- Blood diseases: Coagulopathy or anaemia.
- Infection at the site of injection.
- Drug allergy.

Sampling Method:

Computer generated random number lists.

- Ethical Considerations:

The procedure will be done under supervision of the main supervisor and by an expert. The study will be performed after approval of the Research Ethical Committee of the Faculty of Medicine, Ain Shams University and informed written consent from the parents. The study protocol will be explained to the parents (care givers) before taking their consent to participate in the study and the type of anaesthesia and surgical procedure.

- Study Procedures:

1-Pre-operative settings:

Detailed preoperative history will be taken from the parents of the child for any medical disorder, physical examination and preoperative investigations (complete blood picture, Bleeding Time, prothrombin time and partial

thromboplastin time) will be performed. Age and weight will be recorded. The patient will be fasting according to guidelines preoperatively.

2-Intra-operative Settings

Inside the operating room, mean arterial blood pressure (MAP), heart rate (HR), electrocardiography (ECG) and peripheral oxygen saturation (SpO2) will be obtained using standard non-invasive monitors and base line values will be recorded.

General anesthesia will be induced using face mask with sevoflurane (4-8%) in 80% oxygen, then an intravenous (IV) cannula will be secured. Patients aged (1-4) years an One µg/kg fentanyl will be given and intubation will be facilitated by 0.5 mg/kg atracurium, While Patients aged 4-6 years will receive fentanyl 1 µg/kg, propofol 1-2 mg/kg and intubation will be facilitated by 0.5 mg/kg atracurium. Isoflurane 1%-2% with oxygen was used for anaesthetic maintenance. After stabilizing the patient's hemodynamics and before skin incision, the blocks will be done. (Ozen and Yigit, 2020)

Groups:

The eligible patients will be enrolled in this double blinded study and randomly allocated into one of two parallel equal groups (15 patients in each group) according to sample size by computer generated random number lists. The allocation will be concealed using closed opaque envelopes. Both the patient and the investigator who collected the data after the block will be blind to the study group.

- Multifidus plane block (MPB) group(n=15): Participants will receive injection volume of 1 ml.kg-1 of 0.25% bupivacaine at the fascial plane between the multifidus muscle and the median sacral crests of S2 and S3. **(Suresh *et al.*, 2018; Mahajan *et al.*, 2021)**
- Caudal block (CB) group (n=15): Participants will receive 1 ml.kg−1 of 0.25% bupivacaine, between the two sacral cornua. **(Campbell, 1933; Suresh *et al.*, 2018)**

Block Technique:

- MPB group;

The child will be turned prone and the sacral area will be prepared and sterilized. A linear ultrasound probe (2.5-7.5MHZ) of ultrasound machine (Sonosite turbo M, Bothell, Washington, USA) will be used. Targeting the fascial plane between the multifidus muscle and the median sacral crests of S2 and S3. The block needle is inserted using the "in-plane" technique.

-CB group

- In the left lateral position, CB will be done.

- The ultrasound transducer was first placed transversely at the midline to obtain the transverse view of sacral hiatus. At this level, the ultrasound transducer is rotated 90 degrees to obtain the longitudinal view of sacral hiatus. The block needle is inserted using the "in-plane" technique.
- After completing the block injection, the patient will be immediately placed in the supine position, IV Ringer's solution 10 ml/kg/hour will be administered throughout the surgery.

Potential complications of the blocks:

1- Haemodynamic/systemic or local adverse events. Examples include arrhythmia, hypotension when combined with general anaesthesia.
2- Systemic toxic events from local anaesthetics may involve cardio- or neurotoxicity. Current guidelines recommend that any haemodynamic deterioration should be treated by Intralipid 20% as first-line therapy
3- Toxicity-related seizures that need to be treated with propofol, benzodiazepines, or barbiturates.
4- Infection/inflammation of the puncture site, sacral osteomyelitis, or local nerve injury which can be avoided by good sterilization before the block.
5- Inability to place the block and block failure, so children are generally sedated to ensure immobility during the puncture.
6- Local nerve injury, During MPB care must be taken not to inject directly into the posterior sacral foraminal, which lie lateral to the intermediate sacral crests.

Measurements:

The primary outcome of the study is to use the first time to rescue analgesia postoperative analgesic after two different block techniques to compare the analgesic effect.

The secondary outcome is to compare the two methods for;

- Hemodynamic parameters.
- The total intraoperative IV fentanyl requirements in µg.
- postoperative FLACC pain score and complications.

-Intraoperative:

- Hemodynamic parameters (heart rate and mean arterial pressure) and SpO2 will be measured and recorded just after performing the block and at 15 min interval till the end of the surgery.
- The total intraoperative IV fentanyl requirements in µg, anesthesia time in minutes (from induction of anesthesia till awake extubation), and surgical time in minutes (from skin incision until skin closure) will be recorded.

After the end of surgery, reversal of the atracurium will be done by giving neostigmine in a dose of 0.04 mg/kg and atropine in a dose of 0.02 mg/kg then awake extubation of the patients was done.

3- Post-operative settings:

- FLACC pain score

FLACC is a behavioural pain assessment scale used for nonverbal or preverbal patients who are unable to self-report their level of pain. Pain is assessed through observation of 5 categories including face, legs, activity, cry, and consolability.

- The obtained score will be recorded by a blinded investigator immediately after recovery, then at 2-4-6-12-18 and 24 hours postoperatively.
- Time to first analgesic rescue will be calculated in hours from the recovery of the patient till the first analgesic request (FLACC score ≥ 3).
- Duration of analgesia in hours after awake extubation until the first analgesic request; including diclofenac sodium suppository in a dose of 0.5 mg/kg and the incidence of any side effects such as respiratory depression, urine retention, hematoma, nausea, vomiting, nerve injury, or infection will be recorded. (Joseph et al., 2011)

- Statistical Methods: The collected data will be revised, coded, tabulated, and introduced to a PC using SPSS software version 22. Data will be presented, and suitable analysis will be done according to the type of data obtained for each parameter. Data will be tested first for normality.

1-Descriptive statistics:

- Mean, standard deviation (SD) and range for parametric numerical (quantitative) data.
- Median and inter-quartile range (IQR) for non-parametric data.
- Frequency and percentage of categorical (qualitative) data

2-Analytical statistics:

- Independent-samples t-test of significance was used when comparing between two means.
- Chi-square (X) test of significance was used in order to compare proportions between two qualitive parameters.
- Mann Whitney U test: for two-group comparisons in non-parametric data.
- The confidence interval was set to 95% and the margin of error accepted was set to 5%. So, the p-value was considered significant as the followings:

• Probability (P-value):

P-value <0.05 was considered significant.

P-value <0.001 was considered as highly significant.

P-value >0.05 was considered non_significant.

4. REFERENCES

1. Aksu, C. and Gürkan, Y. (2020) 'Sacral Erector Spinae Plane Block with longitudinal midline approach: Could it be the new era for pediatric postoperative analgesia?', *Journal of Clinical Anesthesia*. Elsevier, 59, pp. 38–39. doi: 10.1016/J.JCLINANE.2019.06.007.
2. Campbell, M. F. (1933) 'Caudal Anesthesia in Children1', *The Journal of Urology*. Wolters KluwerPhiladelphia, PA, 30(2), pp. 245–250. doi: 10.1016/S0022-5347(17)72472-X.
3. Donaire, A. E. and Mendez, M. D. (2023) 'Hypospadias', *StatPearls*. StatPearls Publishing. Available at: https://www.ncbi.nlm.nih.gov/books/NBK482122/ (Accessed: 23 February 2024).
4. Elbadry, A. A. *et al.* (2023) 'Analgesic effect of sacral erector spinae, penile and caudal block after hypospadias surgery: A randomized single blind controlled trial', *Egyptian Journal of Anaesthesia*. Taylor & Francis, 39(1), pp. 563–570. doi: 10.1080/11101849.2023.2235149.
5. Folino, T. B. and Mahboobi, S. K. (2023) 'Regional Anesthetic Blocks', *StatPearls*. StatPearls Publishing. Available at: https://www.ncbi.nlm.nih.gov/books/NBK563238/ (Accessed: 20 January 2024).
6. Forero, M. *et al.* (2016) 'The Erector Spinae Plane Block: A Novel Analgesic Technique in Thoracic Neuropathic Pain', *Regional Anesthesia & Pain Medicine*. BMJ Publishing Group Ltd, 41(5), pp. 621–627. doi: 10.1097/AAP.0000000000000451.
7. Hassan, P. F., Hassan, A. S. and Elmetwally, S. A. (2018) 'Caudal Analgesia for Hypospadias in Pediatrics: Comparative Evaluation of Adjuvants Dexamethasone and Dexmedetomidine Combination versus Dexamethasone or Dexmedetomidine to Bupivacaine: A Prospective, Double-Blinded, Randomized Comparative Study', *Anesthesia, Essays and Researches*. Wolters Kluwer -- Medknow Publications, 12(3), p. 644. doi: 10.4103/AER.AER_77_18.
8. Joseph F. Standing; Dick Tibboel; Reijo Korpela; Klaus T. Olkkola (2011). Diclofenac pharmacokinetic meta-analysis and dose recommendations for surgical pain in children aged 1–12 years. , 21(3), 316–324. doi:10.1111/j.1460-9592.2010.03509.x
9. Mahajan, R. *et al.* (2021) 'Ultrasound-guided sacral multifidus plane block for analgesia following excision of sacrococcygeal teratoma in two neonates', *Anaesthesia Reports*. Wiley-Blackwell, 9(1), p. 81. doi: 10.1002/ANR3.12116.
10. Mistry, T. *et al.* (2022) 'Ultrasound-guided sacral multifidus plane block for sacral spine surgery: A case report', *Saudi Journal of Anaesthesia*. Wolters Kluwer -- Medknow Publications, 16(2), p. 236. doi: 10.4103/SJA.SJA_723_21.
11. Ozen, V., & Yigit, D. (2020). Caudal epidural block versus ultrasound-guided dorsal penile nerve block for pediatric distal hypospadias surgery: A prospective, observational study. Journal of Pediatric Urology, 16(4), 438.e1–438.e8. doi:10.1016/j.jpurol.2020.05.009
12. *Sacral Multifidus Plane/Sacral ESP Block — BABY BLOCKS* (no date). Available at: https://www.baby-blocks.com/block-detail/sacral-multifidus-erector-spinae-plane-block (Accessed: 23 February 2024).
13. Suresh, S. *et al.* (2018) 'The European Society of Regional Anaesthesia and Pain Therapy/American Society of Regional Anesthesia and Pain Medicine Recommendations on Local Anesthetics and Adjuvants Dosage in Pediatric

Regional Anesthesia', *Regional anesthesia and pain medicine*. Reg Anesth Pain Med, 43(2), pp. 211–216. doi: 10.1097/AAP.0000000000000702.
14. Tulgar, S. *et al.* (2019) 'A new technique for sensory blockage of posterior branches of sacral nerves: Ultrasound guided sacral erector spinae plane block', *Journal of Clinical Anesthesia*. Elsevier, 57, pp. 129–130. doi: 10.1016/J.JCLINANE.2019.04.014.
15. Wiegele, M., Marhofer, P. and Lönnqvist, P. A. (2019) 'Caudal epidural blocks in paediatric patients: a review and practical considerations', *BJA: British Journal of Anaesthesia*. Elsevier, 122(4), p. 509. doi: 10.1016/J.BJA.2018.11.030.

The study obtained ethical approval from the Research Ethics Committee, Faculty of Medicine, Ain Shams University, Egypt, (FAMASU REC) which organized and operated according to guidelines of international council on harmonization(ICH) and the Islamic organization of medical sciences(IOMS) , the united states office for human research protections and united state code of federal regulations and operates under federal wide assurance no.FWA000017585

FMASU MD73/2024

Signed by Professor Fathy Tash chair of the REC on 2/5/2024

Faulty of medicine, Ain Shams University, Abbasia ,Cairo, Egypt

.POSTCODE: 11561

Phone number: 202 26857539

Viced.resreach@med.asu.edu.eg
